# Celluloid. What Is It?

**Published:** 1877-02

**Authors:** 


### CELLULOID,—WHAT ABOUT IT?

Dr. H. S. Chase, in the Missouri Dental Journal for January, says: "After four years trial with celluloid, myself and many of my co-laborers find ourselves disappointed in its use. We make more failures in fits than we did with rubber. We have to grind a great deal, after we think we have a perfect articulation; and we find that the color fails. We find that many of the plates warp so as to be useless. We feel that we would be glad to go back to the use of rubber, if it was not for fighting the patent. We even think we would take out a license and cease the warfare if Mr. Bacon would sell us licenses without asking us to swear that we had not used celluloid.

"During the last four months several young dentists have asked my advice about taking out a rubber license. I advised them to do it both for their peace of mind and the benefit of their patients."

Now we really did not expect such a turn as the above indicates, But after all, there is in regard to almost all subjects a diversity of experience and opinion, but the difference of opinion in reference to the use of celluloid, as a base for artificial teeth is more marked than we anticipated it would be at this stage of its use, and there are such diverse aims that we are unable to reconcile or account for them.

Several, and some of them prominent, men in the profession have expressed the same views as Dr, Chase, viz., that celluloid for the purpose indicated is wholly unreliable—a failure. But this is evidence on one side of the question only.

Is there anything to be said in favor of celluloid. Let us see. Dr. C. R. T. says: "During the last two and a half years I have made about forty dentures of the various kinds as they occur in practice, from plates with one tooth to a complete upper and under set, and quite a number of the latter; these have all been worn from the time of insertion to the present without the least unfavorable symptom, either by warping, disintegrating, changing color, or losing the teeth, except one small lateral incisor, standing alone was displaced. Plates have been lain out of the mouth ten days after being finished without the slightest perceptible warping or change, then placed in the mouth and worn as comfortably as plates of any other material. Used rubber for many years, and regard my work with celluloid more successful than with rubber."

Dr. J. A. R, says, "I have been using celluloid exclusively, the cheaper bases, for two or three years with perfect success. It is more satisfactory in every respect than rubber ever was."

Dr. S. says, "I have been using celluloid for about three years, and fully endorse all Dr. R. says about it."

Now we have the names of more than twenty dentists of irreproachable standing, of from ten to twenty-five years experience in practice, whose testimony to the value of celluloid is similar to that just given.

Now how does it occur that so large a number, as is here referred to, can use a material or process with entire success and satisfaction, and others make an entire failure, and others only a partial success at best?

Can it be that those who are claiming success are falsifying and that too when it would be greatly to their disadvantage to do so? Scarcely. When any one claims that he makes an entire failure with any material or process we are disposed to give full credence to his statements, for he has better knowledge on that particular subject than any one else can have, and besides his interest would lead him to claim the result of all success he might attain. So when any one asserts that he fails in any particular matter, we believe him; and when another says he is entirely successful in the same thing we believe him too, if we have faith in his honesty.

In view of these things will somebody rise and explain?

